# Impact of childhood trauma on functionality and quality of life in HIV-infected women

**DOI:** 10.1186/1477-7525-9-84

**Published:** 2011-09-30

**Authors:** Zyrhea CE Troeman, Georgina Spies, Mariana Cherner, Sarah L Archibald, Christine Fennema-Notestine, Rebecca J Theilmann, Bruce Spottiswoode, Dan J Stein, Soraya Seedat

**Affiliations:** 1 South African Research Chairs Initiative (SARChI), PTSD program, Department of Psychiatry, University of Stellenbosch, Cape Town, South Africa; 2 Department of Psychiatry, University of California San Diego, La Jolla, CA, USA; 3 Department of Radiology, University of California San Diego, La Jolla, CA, USA; 4 Cape Universities Brain Imaging Centre (CUBIC), Cape Town, South Africa; 5 MRC Unit on Anxiety and Stress Disorders, Department of Psychiatry, University of Stellenbosch, Cape Town, South Africa; 6 Department of Psychiatry, University of Cape Town, Cape Town, South Africa

**Keywords:** HIV, Quality of Life, Childhood trauma, Functionality

## Abstract

**Background:**

While there are many published studies on HIV and functional limitations, there are few in the context of early abuse and its impact on functionality and Quality of Life (QoL) in HIV.

**Methods:**

The present study focused on HIV in the context of childhood trauma and its impact on functionality and Quality of Life (QoL) by evaluating 85 HIV-positive (48 with childhood trauma and 37 without) and 52 HIV-negative (21 with childhood trauma and 31 without) South African women infected with Clade C HIV. QoL was assessed using the Quality of Life Enjoyment and Satisfaction Questionnaire (Q-LES-Q), the Patient's Assessment of Own Functioning Inventory (PAOFI), the Activities of Daily Living (ADL) scale and the Sheehan Disability Scale (SDS). Furthermore, participants were assessed using the Center for Epidemiologic Studies Depression Scale (CES-D) and the Childhood Trauma Questionnaire (CTQ).

**Results:**

Subjects had a mean age of 30.1 years. After controlling for age, level of education and CES-D scores, analysis of covariance (ANCOVA) demonstrated significant individual effects of HIV status and childhood trauma on self-reported QoL. No significant interactional effects were evident. Functional limitation was, however, negatively correlated with CD4 lymphocyte count.

**Conclusions:**

In assessing QoL in HIV-infected women, we were able to demonstrate the impact of childhood trauma on functional limitations in HIV.

## Background

South Africa is a country severely affected by the AIDS epidemic, with one of the highest rates of HIV infections in the world [1]. The number of premature AIDS related deaths has risen significantly over the last 10 years from 39% to 75% in 2010 [2], resulting in HIV/AIDS being a major, if not principal contributory factor in the overall rising number of deaths. In 2009, UNAIDS estimated the total number of people in South Africa living with HIV to be 5.7 million [3]. It is well known that South African women are disproportionately affected by the disease. 55% of infections were in women, especially women between the ages of 25 and 29 years old, reflected by an HIV prevalence of approximately 40% for this age group [4].

A women's vulnerability to HIV/AIDS is largely attributable not only to biological factors but also socio-economic inequalities. Gender-based violence (GBV) is a common phenomenon in countries where the prevalence rate of HIV is also high. GBV has been defined as a multifaceted phenomenon and can include physical, sexual and emotional violence and deprivation or neglect [5]. Studies conducted in developing countries such as South Africa and other African countries have reported high rates of GBV in both adults and children. This includes intimate partner violence (IPV), rape, and childhood abuse [5-7]. International studies suggest that one out of every three girls is sexually abused by age 18 in the United States [8], and that high prevalence rates of childhood emotional (51.9%), physical (51.1%), and sexual (41.6%) abuse have been reported in HIV-positive individuals [9]. Alarmingly high rates of GBV and revictimisation have been reported in South African women [10-12]. Of 1367 males and 1415 females recruited from 70 rural South African villages, high rates of adverse childhood experiences were documented before the age of 18. The adverse childhood experiences were as follows: physical punishment (89.3% and 94.4%), physical hardship (65.8% and 46.8%), emotional abuse (54.7% and 56.4%), emotional neglect (41.6% and 39.6%), and sexual abuse (39.1% and 16.7%) [12]. In light of the alarmingly high rates of both HIV and childhood trauma among South African women, women living with HIV who also have a history of childhood trauma may be especially susceptible to poorer QoL and functionality due to the additive effects of HIV and acute/chronic stress.

QoL can be defined as "the degree to which persons perceive themselves able to function physically, emotionally and socially" [13]. QoL measures the subjective evaluation of multiple domains of life satisfaction. These cover physical, emotional, functional, psychological, social, personal and environmental domains [14-16].

Although access to and use of more highly active antiretroviral therapies has increased over the past few years, HIV infection and long term use of medication is often accompanied by distressing physical symptoms [17-20] and significant social, financial and psychological demands. Psychiatric symptoms and disorders include anxiety, fear, post-traumatic stress disorder (PTSD) [21] and depression [22,23,19]. Significant levels of depression have been documented in the early phases of HIV [24], suggesting that patients may experience extreme psychological distress, while still being physically asymptomatic. Apart from depression being a secondary diagnosis to HIV/AIDS, depressive symptoms measured over time have also been found to be associated with faster progression of the disease after five years [25]. This finding lends credence to the notion that HIV and depression may have reinforcing effects on each other. Stigmatization has been shown to have a detrimental impact on the mental wellbeing of HIV/AIDS patients. Being avoided or treated with exaggerated kindness by family members or awkward social interaction in healthcare settings has been strongly related to psychological adversity in HIV/AIDS [26].

Several variables impact on Quality of Life (QoL) in HIV. Social factors such as lower educational levels or lower income have been shown to be significant determinants of HIV-related symptom presentation and biological markers such as CD4 lymphocyte count, viral load and mortality [19,27]. Employment also seems to be an important variable in QoL, with HIV-infected individuals in full-time employment, experiencing fewer restrictions in functioning, less anxiety and fewer reported HIV-related symptoms, than those who are unemployed [28]. It has been demonstrated that HIV positive women with larger social support networks reported better mental wellbeing and overall QoL [29,30]. This relationship was also documented in women who practiced more self-care behaviors such as following a healthy diet, adequate sleep and exercise and stress management. These findings reflect the importance of a supportive social network and self care in improving and maintaining QoL in women with HIV [30].

Several studies have revealed that women infected with HIV/AIDS report significantly lower Health Related Quality of Life (HRQoL) than men [31-34]. This was true for men and women infected with HIV-1 Clade C, which is also the predominant viral clade in South Africa [34]. Despite antiretroviral treatment, this effect was still present over time and proved specifically stable in the domains of physical functioning, pain and fatigue [33]. The gender difference in self reported QoL could be attributed to the higher prevalence of mood, anxiety, and somatoform disorders in women [35]. Clear gender differences in HIV progression have also been demonstrated, with women demonstrating a more rapid CD4 cell count decline over time than men [36].

Several studies have investigated the relationship between previous stress, specifically childhood trauma and HIV [37,30,39]. Experiences of violence in childhood, sexual abuse and parental loss have been shown to be significantly associated with an increase in HIV-related risk behaviors in adulthood [40,41]. Specifically, childhood abuse and growing up in unhealthy or unstable environments, could lead to substance abuse, multiple sexual partners, and lack of self-protection - all risk factors for HIV [42-46]. Notably, among African American women who were HIV positive, those who had been traumatized were more likely to meet AIDS criteria than HIV positive women without such a history [38]. Past life trauma not only influences risk behavior, but can also have physiological effects once a person becomes infected [38]. A history of trauma, especially when associated with PTSD, was related to a greater decrease in the CD4/CD8 ratio in HIV infected women compared with non-traumatized HIV infected women [38]. Moreover, a history of childhood physical abuse was associated with higher lifetime rates of major depressive disorder and drug abuse/dependence. This association was especially strong for women [47].

Improvements in HIV treatment, greater availability of medication and an increase in lifespan have led to a greater emphasis on QoL in HIV infected individuals. With the greater availability of antiretroviral treatments in the public health sector, individuals with HIV can expect to live longer lives and pursue normal activities of daily living such as recreation, having social relations and procreation. While many studies have been conducted on HIV and functional limitations, there are very few that examined HIV and early abuse and its combined impact on functionality, highlighting the importance of this study. The current study investigated the specific relationship of childhood trauma on QoL in HIV-infected women. The sample consisted of HIV-positive and HIV-negative women, as well as trauma exposed and non-trauma exposed women. We hypothesized, firstly, that both HIV status and a history of childhood trauma would result in poorer QoL in this sample of women and, secondly, that an interactional effect between HIV status and childhood trauma would be evident, resulting in more severe functional limitations.

## Methods

### Participants

A total of 137 women tested for HIV status were included. 85 were HIV-positive, 48 with childhood trauma and 37 without (from here out referred to as HIV+/trauma + and HIV +/trauma - groups) and 52 were HIV-negative, 21 with childhood trauma and 31 without (from here out referred to as HIV-/trauma + and HIV-/trauma - groups). Although this paper focuses on the QoL and self-perceived functioning of these women, the assessments were part of a larger neurocognitive and neuroimaging study in HIV.

Eligibility criteria included: (I) willingness and ability to provide written informed consent, (II) ability to read and write in either English or Afrikaans at 5^th ^grade level, (III) age between 18 and 65 years, (IV) medically well enough to undergo neuropsychological testing and MRI scanning. Exclusions were: a current or past history of schizophrenia, bipolar disorder or other psychotic disorders as defined by the MINI-plus [48] history of substance or alcohol abuse or dependence as determined on the AUDIT [49], significant previous head injury, demonstrated cognitive impairment on the HIV Dementia Scale, current seizure disorders of any cause, history of CNS infections or neoplasms, hepatitis B positive status, and current use or use within the past month of any psychotropic medication (including antidepressants).

### Procedure

The study was approved by the ethics committee of the University of Stellenbosch, South Africa. All the women included in the present study were tested for HIV status at their local health care facility. HIV status was confirmed by means of Enzyme-linked immunosorbent assay (ELISA), before categorising women into HIV-positive and HIV-negative control groups. The participants were recruited through community health care facilities (VCT sites and HIV units) in and around the Cape metropole of South Africa from 2008-2010. All participants were recruited by a researcher or with the help of doctors and adherence counsellors. Recruitment procedures did not differ between the two groups. All participants who consented were screened for eligibility and childhood trauma exposure either in person at their clinic or telephonically. Those who met initial screening criteria subsequently underwent neuromedical, neuropsychiatric, neurocognitive, and neuroimaging assessments at the University of Stellenbosch. The participants were reimbursed for their travel costs to the University on two separate occasions. The Childhood Trauma Questionnaire (CTQ) was used to elucidate trauma exposure and to categorise HIV-positive and HIV-negative women into the trauma and non trauma exposure groups. For the present study, participants were categorised into the non trauma group if they had a score of 25-40 on the CTQ. Participants were regarded as victims of childhood trauma if they had a score of 41 or higher (moderate-extreme) on the CTQ.

A total of 147 women were recruited, of these 137 completed assessments for this study. Reasons for declining participation included HIV stigma, lack of interest and work/time obligations. In general, HIV infected participants had more health-related concerns and were more willing and available to participate than controls, who were also significantly younger.

### Measures

#### Demographic and health characteristics

Demographic data comprised age, gender, marital status, ethnicity, years of education and employment status. A comprehensive history was obtained from, and a general physical examination conducted in, all patients. CD4-lymphocyte count and viral load parameters were obtained from blood samples to assess for clinical disease progression.

#### Psychiatric diagnosis

All participants were evaluated for current and lifetime psychiatric disorders using the MINI- International Neuropsychiatric Interview- Plus (MINI-Plus) [50], a structured diagnostic interview for major psychiatric disorders that was administered by a psychologist. Participants were also assessed for depressive symptomatology using the Center for Epidemiologic Studies Depression Scale (CES-D). The CES-D is one of the most commonly used self-report screening tools for depression. It consists of 20 statements with a total score ranging from 0 to 60, with higher scores indicating higher levels of depression (CES-D) [51].

### Childhood trauma

Childhood trauma was assessed using the Childhood Trauma Questionnaire Short Form (CTQ-SF), a 28-item self-report inventory that provides valid screening for histories of abuse and neglect. It assesses five types of maltreatment including, emotional, physical, and sexual abuse, and emotional and physical neglect. These five subscales each consist of 5 items with scores ranging from 5 to 25. A summary score assesses overall trauma with scores ranging from 25 to 125. Higher scores indicate higher levels of childhood trauma (score of 25-31 = no trauma, score of 41-51 = low to moderate, 56-68 = moderate to severe, and 73-125 = severe to extreme) [52]. For the present study, participants were categorised into the "no trauma" group if they had a score of 25-40 on the CTQ. Participants were regarded as victims of childhood trauma if they had a score of 41 or higher on the CTQ.

### Quality of Life (QoL) Self-Report Measures

The primary outcome measure was the Quality of Life Enjoyment and Satisfaction Questionnaire (Q-LES-Q). This is a 93-item self-report measure of the degree of enjoyment and satisfaction experienced by participants in various areas of daily functioning. The questionnaire has eight summary scales that reflect major areas of functioning: physical health, emotions, work, household, school hobbies, social relations and general activities. Scores range from 0-100, where higher scores indicate better QoL [53]. Since the Q-LES-Q is a very elaborate questionnaire in assessing eight different categories and is most often used to reflect general QoL in other studies [54], this test was identified as our primary outcome measure of QoL.

Other secondary outcomes measures included the Sheehan Disability Scale (SDS) [55], the Patient's Assessment of Own Functioning Inventory (PAOFI) [56] and the Activities of Daily Living (ADL) [57] scale. The former is a brief self-report tool in which the patient rates the extent to which work/school, social life and home life/family responsibilities are impaired by his or her symptoms. Answers are rated on a 10-point likert scale, with higher scores indicating greater impairment and disability.

The Patient's Assessment of Own Functioning Inventory (PAOFI) is a 41-item questionnaire in which participants rate themselves on neurobehavioral difficulties in their everyday lives, using a 6-point likert scale (almost never, very infrequently, once in a while, fairly often, very often, and almost always). The scale reflects the frequency with which participants experience difficulties with memory, language and communication, sensory-perceptual motor skills, higher level cognitive and intellectual functions, work and recreation, with higher scores indicating more cognitive difficulties [56].

The ADL assesses functioning in several areas: household care, managing finances, groceries, cooking, transportation, using the telephone, home repairs, shopping (non-food), laundry, medication and work. Each area is graded on the level of independence (independently performed, performed with assistance, unable to perform), with greater declines consistent with greater dependence. A participant meets the diagnosis 'ADL- dependant', when he/she has a decline in at least two of the categories [57].

### Data analyses

Data were analyzed using the Statistical Package for the Social Sciences (SPSS) for Windows, version 18.0 and Statistica, version 10. Basic statistical analyses were conducted, which included descriptive statistics. Spearman correlation coefficients were calculated for all QoL self-report measures and depression scores (CES-D) and clinical disease markers (CD4 lymphocyte count and viral load). Reliability analysis (Cronbach's alpha) was conducted on all self-report measures included in the analyses. Analysis of variance (ANOVA) was conducted to assess for group differences in demographic and clinical characteristics. Separate univariate tests of significance, namely Analysis of Covariance (ANCOVA) were computed for the Q-LES-Q and PAOFI. HIV status (HIV-positive and HIV-negative) and childhood trauma status (trauma and no trauma) were included as predictors. Covariates included: age, education, and depression scores. ANCOVA was used to assess both the individual effects and interactional effects of HIV and childhood trauma on self-perceived QoL. Fisher LSD corrections were applied. Finally, confirmatory multiple regression analysis was performed to assess the predictive power of variables of interest on QoL.

## Results

In 72.9% of the HIV infected women, the year of diagnosis ranged from 1993 to 2009 but the majority were recently diagnosed in 2008, leaving 27.1% with an unknown year of diagnosis. The age of the participants ranged from 18-56 years. The average age was 30.06 (*SD *= 7.3) and the average years of education was 10.76 years (*SD *= 1.2). The majority of HIV-positive women were antiretroviral (ARV) naïve (93.4%). Demographic and clinical characteristics of the sample are provided in Table 1.

**Table 1:** Demographic and clinical characteristics of HIV-positive and HIV-negative women with and without childhood trauma (N = 137)

Demographic variable	HIV+/trauma+ (*n *= 48 )	HIV+/trauma- (*n *= 37 )	HIV-/trauma+ (*n *= 21 )	HIV-/trauma- (*n *= 31 )
Mean age (SD)	31.7 (6.9)	31.9 (7.3)	29.8 (7.9)	25.5 (5.6)
Years of education (SD)	10.5 (1.2)	10.6 (1.3)	10.7 (1.3)	11.4 (1.2)
Marital status (%)				
-Single	64.6	64.9	66.7	77.4
-Married	18.8	27	28.6	19.4
-Living with a partner	4.2	2.7	-	-
-Separated	4.2	5.4	-	3.2
-Divorced	6.3	-	4.8	-
-Widowed	2.1	-	-	-
Ethnicity (%)				
-Black	97.9	94.6	95.2	90.3
-Coloured	2.1	5.4	4.8	9.7
Unemployment (%)	68.8	54.1	61.9	61.3
Mean CD4 Cell Count (SD)	403.9 (261.8)	425.5 (254.3)	N.A.	N.A.
Viral Load (SD)	150222.7 (53351.8)	37645.7 (85859.8)	N.A.	N.A.
Mean Q_LES_Q Score	32.4 (1.0)	37.4 (1.2)	35.3 (1.5)	38.8 (1.3)
Mean SDS score (SD)	10.1 (8.1)	6.4 (5.9)	5.9 (6.2)	4.2 (6.3)
Mean ADL decline score	1.4 (1.9)	.9 (1.5)	.2 (.4)	.7 (1.6)
Mean PAOFI score (SD)	13.7 (8.9)	8.1 (7.0)	8.6 (8.7)	5.1 (5.9)
Mean CES-D score (SD)	21.8 (17.5)	7.9 (11.8)	12.8 (14.5)	6.8 (7.1)
Mean CTQ total (SD)	57.7 (10.6)	31.9 (4.3)	58.5 (13.0)	32.4 (4.1)

N.A. Not Applicable

### Reliability analysis

Cronbach alpha coefficients for all measures ranged from satisfactory to excellent: Q-LES-Q (α = .66), SDS (α = .73), ADL (α = .88), CES-D (α = .95), CTQ (α = .70), and PAOFI (α = .97).

### Group differences in demographic and clinical characteristics

Participant characteristics such as age, years of education, marital status, ethnicity, employment status, mean CD4 cell count and viral load are reported in Table 1. Significant group differences were found for age, level of education and mean CES-D score. The mean age was lower in the HIV-/trauma- group (M = 25.5, SD = 5.6), compared to the HIV+/trauma- (M = 31.9, SD = 7.3) and HIV+/trauma+ (M = 31.7, SD = 6.9) groups. ANOVA revealed a significant group difference for age (F = 6.15, p = < .01). The HIV+/trauma+ group had a lower mean educational level (M = 10.5, SD = 1.2) compared to the HIV-/trauma- controls (M = 11.4, SD = 1.2). ANOVA revealed a significant group difference for education (F = 3.46, p = < .05). In terms of depression status, the HIV+/trauma- group had higher mean depression score (M = 7.9, SD = 11.8) than the HIV-/trauma- group (M = 6.8, SD = 7.1), with the highest mean score in the HIV+/trauma+ group (M = 21.8, SD = 17.5). An ANOVA revealed a significant group difference for mean depression scores (F = 10.3, p = < .01).

### Group differences in childhood trauma

In addition to group differences in childhood trauma exposure (F = 103.3, *p *< .001), analyses by abuse type revealed significant differences between trauma+ and trauma- groups on all five subscales (*p *< .001).

### Correlations between QoL measures and CES-D scores

Spearman correlations were computed to assess the relationship between depression and QoL. Significant negative correlations were found between the CES-D and all QoL self-report measures, suggesting that higher depression scores are associated with poorer quality of life, poorer functional status, increased disability, and more subjective neurobehavioural complaints in this sample of women. These included the Q-LES-Q mean score (r = - .33, p < .001), PAOFI total score (r = - .30, p < .001), SDS total score (r = - .31, p < .001), and the ADL total decline(r = - .24, p < .001).

### Correlations between QoL measures and HIV disease markers

Spearman correlations were computed to assess the relationship between CD 4 lymphocyte count, viral load and QoL in this sample of women. There was a significant negative correlation between CD4 counts and PAOFI scores, namely lower CD4 counts were associated with greater disability and more neurobehavioural complaints. However, no relationships were found between CD4 counts or viral load and other functional status measures.

### Group differences in QoL

Means and standard deviations for QoL measures are reported in Table 1. An analysis of covariance using age, education, and depression (CES-D scores) as covariates was conducted in order to investigate the individual and interactional effects of HIV status and childhood trauma on Q-LES-Q scores (Table 2).

**Table 2:** Analysis of Covariance (N = 137)

Dependent variables	HIV		Childhood Trauma		HIV*Childhood trauma	
	**F**	**p**	**F**	**p**	**F**	**p**

Quality of Life	5.16	0.02	6.82	0.01	0.35	0.56
						
Disability	4.89	0.03	1.39	0.24	0.01	0.96
						
Neurocognitive functioning	7.07	0.01	5.95	0.02	0.01	0.91
						
Activities of daily living	6.16	0.01	0.13	0.72	1.49	0.22
						

### Subjective QoL

ANCOVA revealed that both HIV status and childhood trauma status significantly predicted the Q-LES-Q mean total score. Of the three covariates included (age, education, and depression), only age and depression were significant (p < .001). HIV-positive women scored lower on the Q-LES-Q compared to HIV-negative controls, suggesting that HIV is associated with poorer quality of life. Moreover, trauma exposed women scored lower on the Q-LES-Q compared to non-traumatised controls, suggesting that a history of childhood trauma is associated with poorer quality of life. There was no significant interactional effect of HIV status on childhood trauma (Table 2).

### Subjective neurocognitive complaints

ANCOVA revealed that both HIV status and childhood trauma status significantly predicted the PAOFI total score. Of the three covariates included, only depression was significant (p < .001). HIV-positive women scored higher on the PAOFI compared to HIV-negative controls, suggesting that HIV is associated with more subjective neurocognitive complaints. Moreover, trauma exposed women scored higher on the PAOFI compared to non-traumatised controls, suggesting that a history of childhood trauma is associated with more subjective neurocognitive complaints. However, there was no interactional effect between HIV status and childhood trauma (Table 2).

### Confirmatory regression analysis

Finally, as a means for confirmation, a regression analysis was conducted in order to assess the predictive ability of certain variables on subjective QoL in this sample of women. Here again, the Q-LES-Q was used in this analysis. Predictor variables included: age, education, depression, HIV status, and the CTQ total score. The results suggested that the model could explain 31% of the variance in subjective QoL. Age, depression, HIV status, and the CTQ total score significantly predicted QoL in this sample of women, confirming the results from the ANCOVA (Table 3). A second analysis, using only depression, HIV status and the CTQ total score accounted for 19% of the variance in QoL.

**Table 3:** Summary of Multiple Regression Analysis (N = 137)

DV	Predictor	R^2^	ΔR^2^	β	p
Subjective QoL		0.31	0.28		< .000
(Q-LES-Q)	HIV Status			-2.92	< .05
	Age			0.34	< .000
	Education			0.12	0.79
	Childhood Trauma			-0.09	< .05
	Depression			-0.17	< .000

## Discussion

This study set out to investigate childhood trauma and its impact on functionality and QoL among early stage HIV-infected women. In looking at QoL, we did not find any interactional effects between HIV status and a history of childhood trauma in this cohort of women. We did, however, find evidence for both individual HIV and childhood trauma effects on QoL, thereby confirming our first hypothesis. The results revealed that HIV-positive women and traumatised women scored lower on our primary outcome measure (Q_LES_Q), compared to HIV-negative women and non-traumatised controls. The results also revealed that both HIV and a history of childhood trauma were associated with more subjective neurocognitive complaints. Finally, the results provided evidence that HIV is associated with more disability and impairments in everyday functioning, compared to uninfected women. These findings suggest that South African women who are newly infected and have histories of childhood trauma may be particularly at risk for poorer QoL and more disability/impairments in everyday functioning. This may be exacerbated by a lack of social support and fear of revealing HIV status or history of trauma.

It is notable that the lowest QoL scores (Q-LES-Q) were found for the HIV+/trauma+ group, followed by the HIV-/trauma+ group and next the HIV+/trauma- group. This suggests that a history of childhood abuse has a greater negative impact on life enjoyment and satisfaction, than a positive HIV diagnosis alone, even in women with early disease. A decline in function in the early stages of disease was reported in an earlier South African study, with the majority of the decline in function occurring in WHO stages 1 and 2 [58]. In the current study, early infection must be seen against the backdrop of longer term exposure to early life trauma. Thus, with a mean age of 30.1 years most women had been living with experiences of childhood adversity for over 10 years (at a time when HIV risk was low). As such, childhood trauma can reasonably be said to have preceded infection.

A similar pattern was found for depressive symptomatology. Highest depression scores were found for the HIV+/trauma+ group, followed by the HIV-/trauma+ and HIV+/trauma- groups. This, too, suggests that experience of childhood trauma may have a greater association with depressive symptoms than HIV *per se*, and a positive HIV diagnosis may further strengthen depressive symptomatology. Of note, several studies have reported an association between gender-based childhood trauma, in particular childhood sexual abuse, and HIV risk in later life [59-61]. Childhood trauma may increase HIV risk indirectly by increasing high-risk behaviors or by disabling prevention choices. Childhood trauma is strongly associated with adult revictimization which can further compound the risk for HIV among women [62]. Childhood trauma also presents as a potent antecedent to adult-onset depression, with neuroendocrine changes secondary to early-life stress predisposing to the risk for depression [63]. Depression, once set in, can further impact upon specific elements of immune system functioning in HIV and, through this mechanism, may influence quality of life and health status [64]. What also needs to be taken into account is that individuals living with HIV/AIDS are faced with concealable, yet considerable stigma, discrimination and psychological distress, previously believed to accompany visible stigma's only [65]. Apart from stigmatization's negative impact on various aspects of social life and mental well-being [66,26], Pachankis, stresses that "the ambiguity of social situations combined with the threat of potential discovery, makes possessing a concealable stigma a difficult predicament for many individuals" [65]. Furthermore, AIDS related stigmatization has been shown to inhibit individuals from seeking crucial health-related care, including voluntary HIV testing and counseling [66]. Since both childhood trauma and HIV encompass a great risk for stigmatization and the individual's desire for concealment, having experienced both and taking all other previously mentioned factors into account, could further explain our findings of lower functionality and QoL in the HIV+/trauma+ group.

In terms of virologic status, there was a significant, negative correlation between CD4 counts and PAOFI scores, namely lower CD4 counts were associated with greater disability and more neurobehavioral complaints. However, no relationships were found between CD4 counts or viral load and other functional status measures. While the absolute CD4 count is more predictive of clinical disease progression than viral load [25], single measurements of both CD4 and viral load may be inconsistent and prone to transient and insignificant fluctuations. This may explain why CD4 counts were significantly related to functional limitations while viral loads were not. Lastly, significant correlations were found among all four questionnaires, reflecting a close association between lower degrees of life enjoyment and satisfaction (Q-LES-Q), higher scores of disability (SDS), more functional decline (ADL) and neurocognitive complaints (PAOFI). It also suggests a level of consistency among these four measures on disability/QoL reporting.

A few limitations are worth noting. Firstly, study participants were recruited from health care clinics in one South African province which raises a question about generalisability. However, sample characteristics are largely reflective of the socio-demographic and economic conditions of HIV-infected persons throughout South Africa. In addition, given the variation in years of education among our participants, less literate patients may have encountered more difficulty completing the self-report measures, potentially contributing to response bias. The sample size is relatively small but suitable for the neuroimaging assessments, which was also an aim of the larger study. However, it is worth noting that power is a fundamental issue to consider in conducting an interaction analysis. In light of this, it is plausible that the insignificant interaction effect was due to the relatively small sample size in the present study. Furthermore, CD4 counts and viral loads were only measured at the initial clinical assessment with no serial monitoring. Other limitations include the retrospective assessment of childhood trauma and the fact that this was a cross-sectional study which precludes conclusions to be drawn about causality. Longitudinal investigation of the temporal ordering of depression and QoL deterioration in HIV infected women with early gender-based violence will be key to elucidating these relationships. In addition, HIV-related stigma and disclosure were not taken into account and should be considered in future research.

The present study has mentionable strengths. It is, to our knowledge, the first to assess QoL secondary to childhood trauma in predominantly antiretroviral naïve HIV-infected women compared with their HIV-negative counterparts. In addition, the use of four complementary measures of QoL and disability permitted comprehensive cross-sectional assessment of functionality, rarely evident in the literature. In assessing QoL in a sample of HIV-infected women, this study primarily demonstrates that the experience of childhood trauma can have a greater negative impact on QoL and depressive symptomatology than a positive HIV diagnosis alone. These findings underscore the need to screen for childhood trauma, associated psychopathology and functionality in women and men who are HIV positive and to address these issues in management, even in HIV patients who are still physically asymptomatic. Moreover, the study highlights the need for HIV prevention activities such as education in HIV risk behaviors and an increased focus on identification and support for children and youth who have experienced childhood traumas. It also emphasizes the necessity of early recognition and management of mood, anxiety and other stress-related disorders. Finally our findings reflect the need to help improve and maintain QoL in HIV positive and traumatized individuals [38,67,68]. This includes social support interventions which have the potential not only to improve QoL but also to relieve cognitive symptom and depressive symptom burden [29]. To this effect, an intervention study by Sikkema et al., proved successful in reducing both intrusive and avoidant traumatic stress symptoms, which emphasizes the need for similar interventions in HIV+ trauma victims [69]. Trauma has been associated with poor adherence, poor QoL and shame [70]. Specifically, Cohen et al., and Kang, Goldstein, & Deren, found an association between childhood maltreatment and poor adherence to ARVs [60,71] which demonstrated the need to improve access to and retention on ARVs considering that ARVs are known to have strong positive effects on QoL and improving health status [72,73].

## Conclusion

South African women are disproportionately affected by HIV/AIDS and childhood trauma. In assessing QoL in HIV-infected women, we were able to demonstrate the impact of childhood trauma on functional limitations in HIV. The experience of childhood trauma proved to have a negative impact on QoL and functionality in this cohort of women.

## Competing interests

The authors declare that they have no competing interests.

## Authors' contributions

ZT performed statistical analyses and drafted the manuscript. GS participated in acquisition of data, statistical analyses, its design and coordination and helped to draft the manuscript, MC, SL, CF-N, RT, BS, DS, and SS participated in its design and coordination and helped to draft the manuscript. All authors read and approved the final manuscript.
